# Supplementary material for: A Critical View of the Application of the APEX Software (Aqueous Photochemistry of Environmentally-Occurring Xenobiotics) to Predict Photoreaction Kinetics in Surface Freshwaters
Source: Molecules. 2019 Dec 18;25(1):9. doi: 10.3390/molecules25010009 (PMC7017383; doi:10.3390/molecules25010009)
Supplement: Supplementary file 1 [file molecules-25-00009-s001.zip › 3.2.4_gcc-4.4.0.zip!/3.2.4_gcc-4.4.0/3.2.4_gcc-4.4.0/doc/octave/html/interpreter/Surface-group.html]

Surface group - Untitled


Previous: Stem Series,
Up: Object Groups


---

##### 15.2.8.11 Surface group

Surface group objects are created by the `surf` or `mesh`
functions, but are equally one of the handles returned by the `surfc`
or `meshc` functions. The surface group is of the type `surface`.

The properties of the surface group are

`edgecolor` `facecolor`: The RGB color or color name of the edges or faces of the surface. See Colors. `linewidth` `linestyle`: The line width and style of the lines on the surface. See Line Styles. `marker` `markeredgecolor` `markerfacecolor` `markersize`: The line and fill color of the markers on the surface. See Colors. `xdata` `ydata` `zdata` `cdata`: The original x, y, z and c data. `xdatasource` `ydatasource` `zdatasource` `cdatasource`: Data source variables.
